# Boswellic Acids Show In Vitro Activity against *Leishmania donovani*

**DOI:** 10.3390/molecules26123651

**Published:** 2021-06-15

**Authors:** Hippolyt L. Greve, Marcel Kaiser, Pascal Mäser, Thomas J. Schmidt

**Affiliations:** 1 Institute of Pharmaceutical Biology and Phytochemistry, University of Münster, Pharma Campus–Corrensstrasse 48, D-48149 Münster, Germany; hippolyt.greve@uni-muenster.de; 2 Department of Medical Parasitology and Infection Biology, Swiss Tropical and Public Health Institute, Socinstrasse 57, CH-4051 Basel, Switzerland; marcel.kaiser@swisstph.ch (M.K.); pascal.maeser@swisstph.ch (P.M.); 3 University of Basel, Petersplatz 1, CH-4003 Basel, Switzerland

**Keywords:** *Boswellia serrata*, frankincense, Burseraceae, *Leishmania donovani*, antiprotozoal activity, boswellic acids

## Abstract

In continuation of our search for leads from medicinal plants against protozoal pathogens, we detected antileishmanial activity in polar fractions of a dichloromethane extract from *Boswellia serrata* resin. 11-keto-β-boswellic acid (KBA) could be isolated from these fractions and was tested in vitro against *Leishmania donovani* axenic amastigotes along with five further boswellic acid derivatives. 3-*O*-acetyl-11-keto-β-boswellic acid (AKBA) showed the strongest activity with an IC_50_ value of 0.88 µM against axenic amastigotes but was inactive against intracellular amastigotes in murine macrophages

## 1. Introduction

Vector-borne diseases caused by protozoal pathogens remain a major health problem, especially in low-income countries in sub-tropical and tropical areas [1]. We recently reported results of bioactivity-guided fractionations of Burseraceae resins aiming to find compounds with activity against the malaria parasite *Plasmodium falciparum (Pf)* [2,3]. In continuation of our search for antiprotozoal leads from plants, we also tested extracts and fractions obtained from *Boswellia* and *Commiphora* spp. for their antileishmanial activity. Leishmaniases are classified as a Neglected Tropical Disease (NTD) according to the World Health Organization (WHO). They are endemic in large parts of Africa, (South) America and Asia and, therefore, are a health hazard for millions of people. The causative agents are about 20 human-pathogenic species from the genus *Leishmania* that are transmitted to humans by the bite of infected female sandflies from the genera *Lutzomyia* or *Phlebotomus*. The disease occurs in three different clinical manifestations (cutaneous, mucocutaneous or visceral). The visceral form (“kala-azar”), caused among other species by *L. donovani (Ldon)*, and affecting the inner organs is the most dangerous form. Although axenic culture of *Leishmania* is possible in the laboratory, in mammals, the parasites are obligatory intracellular residing in their amastigote form in the host’s macrophages. This is one reason why present treatment options such as pentavalent antimonials, pentamidine or amphotericin B are problematic due to severe side effects. As developing resistances, as well as the expensiveness of existing therapies, remains a problem, safe and affordable new treatment options are urgently needed [1].

The plant family of Burseraceae contains several members that have been used in ethnopharmacology for centuries. *Boswellia serrata* Roxb. is a deciduous tree native to India with a greyish papery bark. It exudes a fragrant, yellowish oleo-gum-resin, called Indian frankincense or Olibanum, that is valued for its anti-inflammatory properties. The main components of oleo-gum resins are terpenoids, gums and essential oils [4]. Boswellic acids, terpenoid compounds that occur in considerable amounts in the drug, have been shown to contribute to these effects. In our bioactivity-guided search for antiplasmodial compounds, the dichloromethane extract of the oleo-gum resin displayed the highest activity, indicating that terpenoid compounds might be involved. Fractionation of this extract revealed that triterpenoid compounds, in particular, contribute to its antiplasmodial effects [2]. Whereas antimicrobial activities of essential oils are well known in general, reports about the application of *Boswellia* oleo-gum-resin or its constituents against Leishmaniasis are scarce [5,6]. A commercially available essential oil, mainly obtained from *B. carteri* alongside further *Boswellia* spp., showed an IC_50_ value of < 12.5 µg/mL against promastigotes and 22.1 ± 4.2 µg/mL against intracellular amastigotes of *Leishmania amazonensis* [7]. Serratol, a diterpene isolated from *B. serrata*, showed antiplasmodial activity in vitro but was essentially inactive against *L. donovani* axenic amastigotes [8]. A methanolic extract of *B. carteri* resin showed no activity against *L. infantum* amastigotes [9].

## 2. Results and Discussion

Based on a repurposing screening of herbal medicinal products [10] and results from in vitro tests of serratol [8], antiplasmodial effects of different Burseraceae were investigated in a systematical manner. Resins were successively extracted with solvents of increasing polarity (DCM, EtOH, H_2_O). While aqueous extracts did not show any activity, DCM extracts from *B. serrata* and *Commiphora myrrha* appeared most promising for a bioactivity-guided isolation of their constituents active against *Plasmodium falciparum* as reported previously [2,3]. Besides their antiplasmodial effects, the non-aqueous extracts from *B. serrata* and *B. carteri* displayed considerable activity against *L. donovani* as well, whereas they were less active against *Trypanosoma brucei rhodesiense* (*Tbr*) and *T. cruzi (Tc)* (Table 1).

A growth inhibition assay of fractions Bs-1–Bs-20 obtained from the DCM-extract of *B. serrata* by column chromatography on silica [2] showed that antileishmanial activity is associated with increasing polarity of the fractions (Table 2).

**Figure 1:**
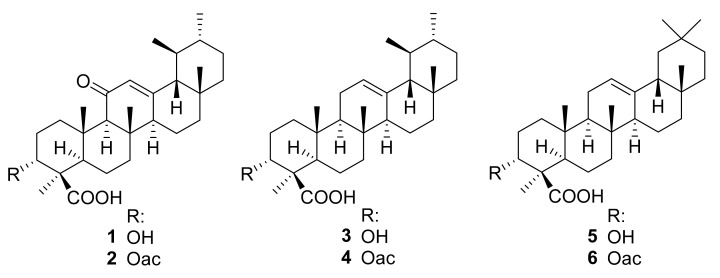
Chemical structures of boswellic acids **1**–**6**.

11-keto-β-boswellic acid (KBA, **1**) could be isolated from these fractions and further boswellic acid derivatives could be determined as constituents of respective fractions by TLC and HPLC analysis in comparison with reference substances (data not shown). Consequently, the IC_50_ values of the isolated KBA, as well as five related boswellic acid derivatives against axenic amastigotes of *L. donovani,* were determined (see Table 3): 3-*O*-acetyl-11-keto-β-boswellic acid (AKBA, **2**), β-boswellic acid (**3**), 3-*O*-acetyl-β-boswellic acid (**4**), α-boswellic acid (**5**), and 3-*O*-acetyl-α-boswellic acid (**6**) (Figure 1).

KBA (**1**) and AKBA (**2**) showed the best results with 1.9 µM and 0.88 µM, respectively, against axenic amastigotes and thus were considerably more active than the congeners lacking the keto group at C-11 (IC_50_ ranging from 2.4 to >6 µM). This indicates that the 11-keto-group increases antileishmanial activity. The positive control miltefosine displayed an IC_50_ value of 0.12 µM in this assay. Most of the tested derivatives showed a similar degree of low cytotoxicity (IC_50_ ranging from 14 to 33 µM) against L6 rat skeletal myoblasts, while KBA (**1**) was even less cytotoxic (IC_50_ = 91 µM). KBA (**1**) and AKBA (**2**) showed the best selectivity indices with values of 46 and 38, respectively (Table 3). Due to its strong activity against axenic amastigotes, AKBA (**2**) was tested against intracellular amastigotes in peritoneal murine macrophages in a further assay, but at a concentration of 10 µg/mL, no activity could be determined. This may be related to the compound’s acidic nature. Intracellular *Leishmania* amastigotes reside within macrophages in parasitophorous vacuoles which provide an acidic environment (pH 4.7–5.2) [11]. It can be expected that carboxylic acids with otherwise non-polar molecular structures such as the compounds under study will be poorly soluble under these conditions so that they may not reach the intracellular parasites. Fusing of parasitophorous vacuoles with phagolysosomes leads to an environment with increased hydrolytic activity. Therefore, the fate of hydrolysable moieties, such as the 3-*O*-acetyl-group, remains unclear [12]. Moreover, it has to be considered that exogenous compounds have to pass multiple membranes until they can come in contact with the amastigote [13]. It will, hence, be interesting to investigate the antileishmanial activity of non-acidic 11-ketotriterpenes and/or steroids. Such studies have been initiated.

## 3. Materials and Methods

The oleo-gum resins were purchased from commercial sources. Resin of *B. serrata* was obtained from Pharmasan GmbH (Freiburg, Germany, batch no. PS0208030), resin of *B. carteri* (“Olibanum in granis”, batch no. 14107801) and myrrh resin (“Myrrha conc.”, batch no. 12269911) from Caesar and Loretz GmbH (Hilden, Germany;). Voucher samples (number PB226, HG_Cm1 and HG_Bc1, resp.) were deposited at the documentation file of the Institute of Pharmaceutical Biology and Phytochemistry, IPBP, University of Münster.

The extraction and fractionation process leading to the fractions tested in this study (Table 2) and to the isolation of 11-keto-β-boswellic acid (KBA, **1**), including all experimental details, is described in our previous communication [2]. Briefly, 125 g of powdered *Boswellia serrata* resin was extracted exhaustively using DCM in a Soxhlet apparatus. Then, 25 g of the 78 g yielded was fractionated by column chromatography on 1.3 kg silica (7.5 × 80 cm) using a step gradient of a hexane:ethyl acetate mixture (98:2—3.7 L; 95:5—12.4 L; 90:10—5.2 L; 80:20—3.4 L; 70:30—4.05 L; 50:50—4.35 L; 0:100—4.5 L). Elution volumes and yields of the 20 resulting fractions were as follows: Bs-1: 2190–3900 mL, 158 mg; Bs-5: 10,250–11,780 mL, 268 mg; Bs-7:12,650–13,560 mL, 48 mg; Bs-13: 22,670–23,460 mL, 463 mg; Bs-15:24,930–27,030 mL, 486 mg; Bs-17: 28,150–29,580 mL, 1344 mg; Bs-20: 32,440–37,560 mL, 5270 mg. In total, 200 mg of fraction Bs-20 was purified with a Jasco HPLC system on a Reprosil 100 C-18 column (5 µm, 250 × 20 mm) using a methanol:water gradient (start—70:30; 8–25 min—100:0; 9 mL/min: t_R_ = 17–18 min) to yield 6.3 mg of KBA (**1**). 3-*O*-acetyl-11-keto-β-boswellic acid (AKBA, **2**), β-boswellic acid (**3**), 3-*O*-acetyl-β-boswellic acid (**4**), α-boswellic acid (**5**), and 3-*O*-acetyl-α-boswellic acid (**6**) were kindly provided by Phytolab GmbH & Co. KG, (Vestenbergsgreuth, Germany). The purity of the compounds was >95%.

In vitro tests for determination of activity against axenic amastigotes of *Leishmania donovani* (MHOM-ET-67/L82 strain) and cytotoxicity against mammalian cells (L6-cell-line from rat skeletal myoblasts) as well as against intramacrophage amastigotes of *L. donovani* were conducted at Swiss TPH according to an established protocol as reported before [14,15]. For a repetition of these methods, see Supplementary File.

Miltefosine (≥98%, Sigma-Aldrich, Buchs, Switzerland) and podophyllotoxin (≥98%, Sigma-Aldrich, Buchs, Switzerland) were used as positive controls. Growth inhibition assays were meant to give only preliminary information on whether fractions show any bioactivity or not. Due to this explorative nature, this was conducted once. IC_50_ values represent geometric means of two independent determinations and were calculated by linear regression [16] from the sigmoidal dose inhibition curves using SoftmaxPro (Molecular Devices Cooperation, Sunnyvale, CA, USA) software.

## Figures and Tables

**Table 1:** Bioactivity data (IC_50_ values in µg/mL) of extracts from selected Burseraceae resins. Results of double determinations are given as geometric mean; maximum and minimum values are given in parentheses. Further values are results from single determinations.

	*Tbr*	*Tc*	*Ldon*	*Pf*	L6
***Boswellia serrata***					
DCM	12	15 (14; 17)	4.5 (3.9; 5.1)	2.6 (2.4; 2.8)	46 (45; 47)
EtOH	11 (9.3; 11)	23 (20; 26)	4,7 (4.6; 4.9)	3.4 (2.9; 3.9)	54 (54; 54)
H_2_O	45	65	> 100	> 50	> 100
***Boswellia carteri***					
DCM	14	12 (9.9; 15)	3.0 (2.4; 3.8)	3.4 (2.4; 4.7)	39 (37; 42)
EtOH	15	14 (12; 17)	3.0 (2.4; 3.7)	3.8 (3.2; 4.4)	44 (43; 45)
H_2_O	34	77	> 100	> 50	> 100
***Commiphora myrrha***					
DCM	5.2 (5.1; 5.4)	16 (12; 21)	6.1 (5.5; 6.7)	1.0 (1.0; 1.0)	8 (6; 11)
EtOH	13	41	15	1.8 (1.2; 2.6)	42 (39; 46)
H_2_O	45	61	> 100	> 50	> 100

**Table 2:** Results from activity tests of fractions (Bs-1–Bs-20) of increasing polarity obtained by column chromatography of a DCM extract from *B. serrata* resin. Each value represents growth inhibition in %, in comparison to untreated control. As these assays were meant to give only preliminary information on whether fractions show any bioactivity or not, they have only been conducted once. Fractions Bs-2 and Bs-18 were not tested because of strong similarity of their chemical profiles to neighboring fractions.

Fraction	*L. donovani*	
	10 µg/mL	2 µg/mL
Bs-1	31	0.0
Bs-3	0.0	0.0
Bs-4	1.0	0.0
Bs-5	79	0.0
Bs-6	76	0.0
Bs-7	21	0.0
Bs-8	21	0.0
Bs-9	85	31
Bs-10	47	25
Bs-11	51	27
Bs-12	42	24
Bs-13	70	28
Bs-14	61	25
Bs-15	81	31
Bs-16	89	30
Bs-17	100	41
Bs-19	100	36
Bs-20	100	37

**Table 3:** In vitro activity data of boswellic acid derivatives against amastigote forms of *Leishmania donovani*; values represent geometric means of two independent determinations, the lower and upper values are reported in parentheses.

No.	*Leishmania donovani* Axenic Amastigotes		Cytotoxicity against L6 Rat Skeletal Myoblasts		
	IC_50_ (µg/mL)	IC_50_ (µM)	IC_50_ (µg/mL)	IC_50_ (µM)	SI
**1**	0.9 (0.51; 1.7)	1.9	43 (42; 45)	91	46
**2**	0.45 (0.37; 0.54)	0.88	17 (17; 18)	33	38
**3**	2.4 (1.5; 3.7)	5.3	15 (14; 16)	33	6.2
**4**	6.1 (3.1; 12)	12	16 (16; 17)	32	2.7
**5**	n.a. (2.3; >100)	n.a.	14 (14; 15)	31	n.a.
**6**	3.1 (1.4; 6.9)	6.2	7.2 (6.4; 8.0)	14	2.3
**PC_M_^a^**	0.047 (0.035; 0.064)	0.12	n.a.	n.a.	n.a.
**PC_P_^b^**	n.a.	n.a.	0.009 (0.007; 0.011)	0.02	n.a.
	**Intracellular Amastigotes ^c^**		**Cytotoxicity against** **Macrophages**		
**2**	> 10	n.a.	20 (20; 20)	39	n.a.
**PC_M_^a^**	1.9 (2.6; 1.4)	4.7	n.a.	n.a.	n.a.

SI: selectivity index; ^a^ positive control: miltefosine; ^b^ positive control: podophyllotoxin; ^c^ in peritoneal murine macrophages; n.a. = not available.

## Data Availability

Data reported in this study is contained within the article and supplementary material. The underlying raw data is available on request from the corresponding author.

## Supplementary Materials

Experimental details and dose-effect diagrams from the biological tests of the compounds for antileishmanial and cytotoxic activity are available as Supporting Information (Figures S1–S3).

## Sample Availability

Samples of the compound **1** are available from the authors.
