# Supplementary material for: Boswellic Acids Show In Vitro Activity against Leishmania donovani
Source: Molecules. 2021 Jun 15;26(12):3651. doi: 10.3390/molecules26123651 (PMC8232742; doi:10.3390/molecules26123651)
Supplement: Supplementary file 1 [file molecules-26-03651-s001.zip › molecules-1228808-supplementary.pdf]

Supplementary Materials

Boswellic Acids Show In Vitro Activity against *Leishmania donovani*

Hippolyt L. Greve [1], Marcel Kaiser [2,3], Pascal Mäser [2,3] and Thomas J. Schmidt [1,*]

[1] Institute of Pharmaceutical Biology and Phytochemistry, University of Münster, Münster, Germany; hippolyt.greve@uni-muenster.de(H.L.G.); thomschm@uni.muenster.de (T.J.S,)
[2] Department of Medical Parasitology and Infection Biology, Swiss Tropical and Public Health Institute, Basel, Switzerland; pascal.maeser@swisstph.ch (P.M.); marcel.kaiser@swisstph.ch (M.K.)
[3] University of Basel, Basel, Switzerland
* Correspondence: thomschm@uni-muenster.de; Tel.: +49 251 8333378

Biological Assays (according to [14,15])

The following biological tests were performed by applying validated methods of the Swiss TPH as previously published [14] and described below.

In vitro activity against *Trypanosoma brucei rhodesiense*. Minimum Essential Medium (50 μL), supplemented with 25 mM HEPES, 1 g/L additional glucose, 1% MEM non-essential amino acids (100×), 0.2 mM 2-mercaptoethanol, 1mM Na-pyruvate, and 15% heat inactivated horse serum, was disposed in 96-well microtiter plates. Serial compound dilutions of eleven 3-fold dilution steps covering a range from 100 to 0.002 μg/mL were prepared. Then, an aliquot containing $4x10^3$ bloodstream forms (trypomastigotes) of *T. b. rhodesiense* STIB 900 in 50 μL were added to each well, and the plate was incubated at 37 °C under 5% CO_2 atmosphere for 70 h. After that, resazurin solution (12.5 mg in 100 mL water, 10 μL) was added to each well, and the incubation continued for 2–4 h more. Then, the plates were read using a Spectramax Gemini XS microplate fluorometer (Molecular Devices Cooperation, Sunnyvale, CA, USA) and excitation and emission wavelengths of 536 and 588 nm, respectively. Data were analyzed with the software Softmax Pro (Molecular Devices Cooperation, Sunnyvale, CA, USA), which calculated IC_{50} values by linear regression and 4-parameter logistic regression from the sigmoidal dose inhibition curves. Melarsoprol was used as positive control.

In vitro activity against *T. cruzi*. Rat skeletal myoblasts (L-6 cells) were seeded in 96-well plates at 2000 cells/well in 100 μL RPMI 1640 medium with 10% FBS and 2 mM L-glutamine. After 24 h, the medium was removed and replaced by 100 μL per well containing 5000 trypomastigote forms of *T. cruzi* Tulahuen strain C4 containing the β-galactosidase (Lac Z) gene. After 48 h, the medium was removed from the wells and replaced by 100 μL fresh medium with or without a serial compound dilution (as mentioned for *T. brucei rhodesiense*). After 96 h of incubation, the plates were inspected under an inverted microscope to assure growth of the controls and sterility. Then, substrate CPRG/Nonidet (50 μL) was added to each well. The color reaction was developed within 2–6 h, and the plate read at 540 nm. Data were analyzed as mentioned in the previous section. Benznidazole was used as positive control.

In vitro activity against *L. donovani* axenic amastigotes. Amastigotes of *L. donovani* strain MHOM/ET/67/L82 were grown in axenic culture at 37 °C in SM medium at pH 5.4 supplemented with 10% heat-inactivated fetal bovine serum under a 5% CO_2 atmosphere. Aliquots containing 10^5 amastigotes from the axenic culture (100 μL), with or without a serial compound dilution (as specified before), were seeded in 96-well plates. After 70 h of incubation, the plates were inspected under an inverted microscope to assure growth of the controls and sterile conditions. Then, resazurin solution (12.5 mg in 100 mL water, 10 μL) was added to each well and the plates incubated for another 2 h. Finally, the plates were read using the same conditions mentioned for *T. brucei rhodesiense*. Data were analyzed in the same way. Miltefosine was used as positive control.

In vitro activity against *P. falciparum*. In vitro activity against erythrocytic stages of *P. falciparum* was determined using a 3H-hypoxanthine incorporation assay, employing the drug-sensitive NF54 strain (Schipol Airport, The Netherlands). Compounds were diluted with medium as indicated above before being added to parasite cultures incubated in RPMI 1640 medium without hypoxanthine, supplemented with HEPES (5.94 g/L), $NaHCO_3$ (2.1 g/L), neomycin (100 U/mL), AlbumaxR (5 g/L) and washed human red cells A+ at 2.5% hematocrit (0.3% parasitemia). Serial compound dilutions were prepared as before. The 96-well plates were incubated in a humidified atmosphere at 37 °C; 4% CO_2, 3% O_2, 93% N_2. After 48 h, 50 μL of 3H-hypoxanthine (0.5 μCi) was added to each well of the plate. The plates were incubated for a further 24 h under the same conditions. The plates were then harvested with a Betaplate™ cell harvester (Wallac, Zurich, Switzerland), and the red blood cells were transferred onto a glass fiber filter then washed with distilled water. The dried filters were inserted into a plastic foil with 10 mL of scintillation fluid and counted in a Betaplate™ liquid scintillation counter (Wallac, Zurich, Switzerland). Data were treated as described above. Chloroquine and artemisinin were used as positive controls.

In vitro cytotoxicity with L-6 cells. Assays were performed in 96-well plates. Each well was seeded with 100 μL of RPMI 1640 medium supplemented with 1% L-glutamine (200 mM), 10% fetal bovine serum, and 4000 L-6 cells. Serial compound dilutions prepared, as before, were used. After 70 h of incubation, the plates were inspected under an inverted microscope to assure growth of the controls and sterile conditions. Resazurin solution (12.5 mg in 100 mL water, 10 μL) was then added to each well and the plates incubated for another 2 h. Finally, the plates were read, and the data were treated as mentioned before. Podophyllotoxin was used as positive control.

In addition, tests for in vitro activity against intracellular amastigotes of *L. donovani* were performed as follows (according to [15]):

1×10^4 murine peritoneal macrophages were seeded in 100 μL RPMI 1640 medium with 10 % FBS in 96-well-plates. After 24 h, they were infected with 2×10^5 axenic amastigotes (cultured at pH 5.4 as above) in 100 μL of medium. Extracellular amastigotes were removed by triplicate washing after further 24 h. Subsequently, the dilutions of the test compounds were added. After incubation for 96 h, the medium was removed. The plate was then fixed with 4% formaldehyde stained with the DNA-dye DRAQ5. Images of the plates were recorded with an ImageXpress XLS (MD) microscope and automatically analyzed using the MetaXpress Software. Infected and non-infected macrophages, as well as the number of intracellular parasites, were recorded. Thereby, the infection rate, as well as the reduction in the number of intracellular amastigotes, can be determined in comparison with an untreated control.

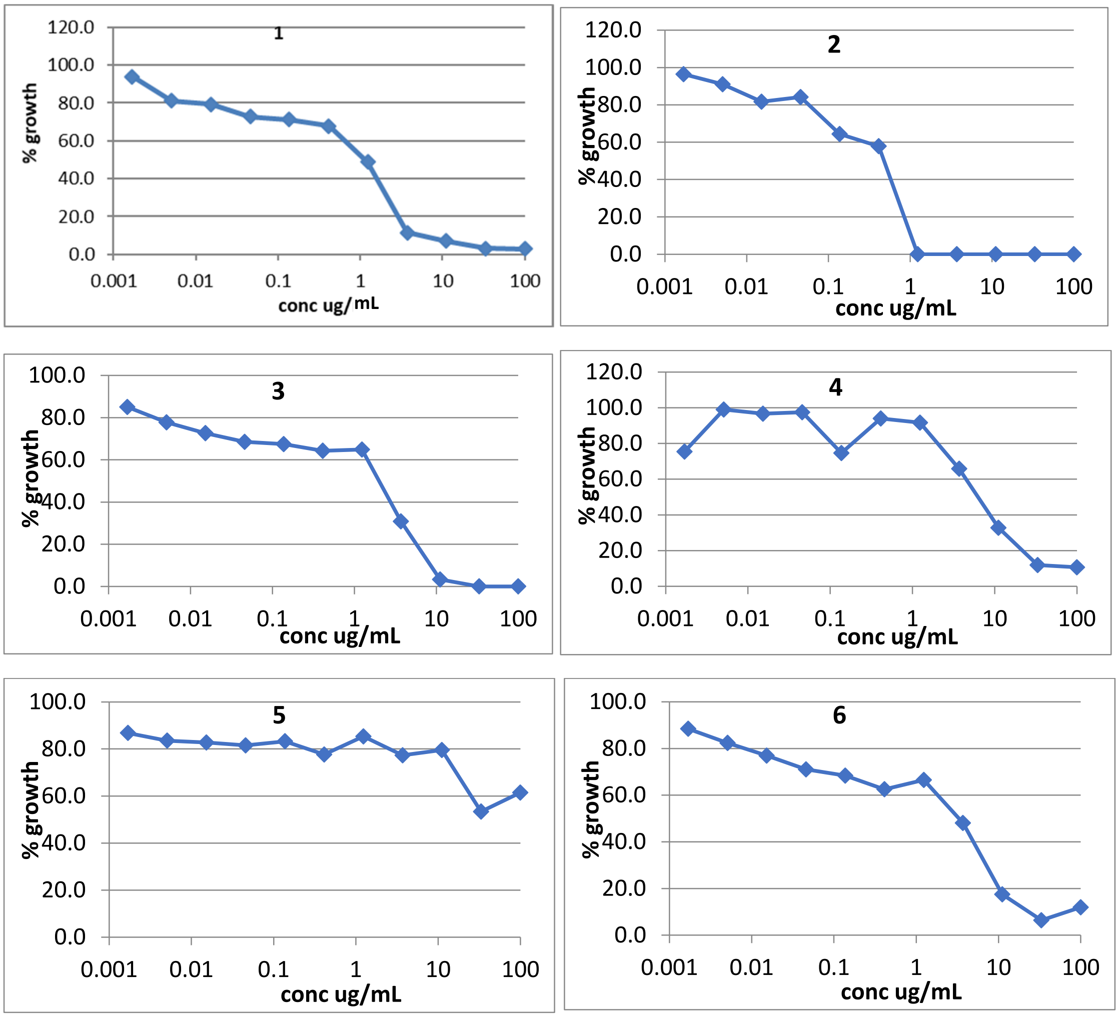

Figure S1: Dose–effect diagrams for bioactivity of compounds **2–6** against *Leishmania donovani* axenic amastigotes.

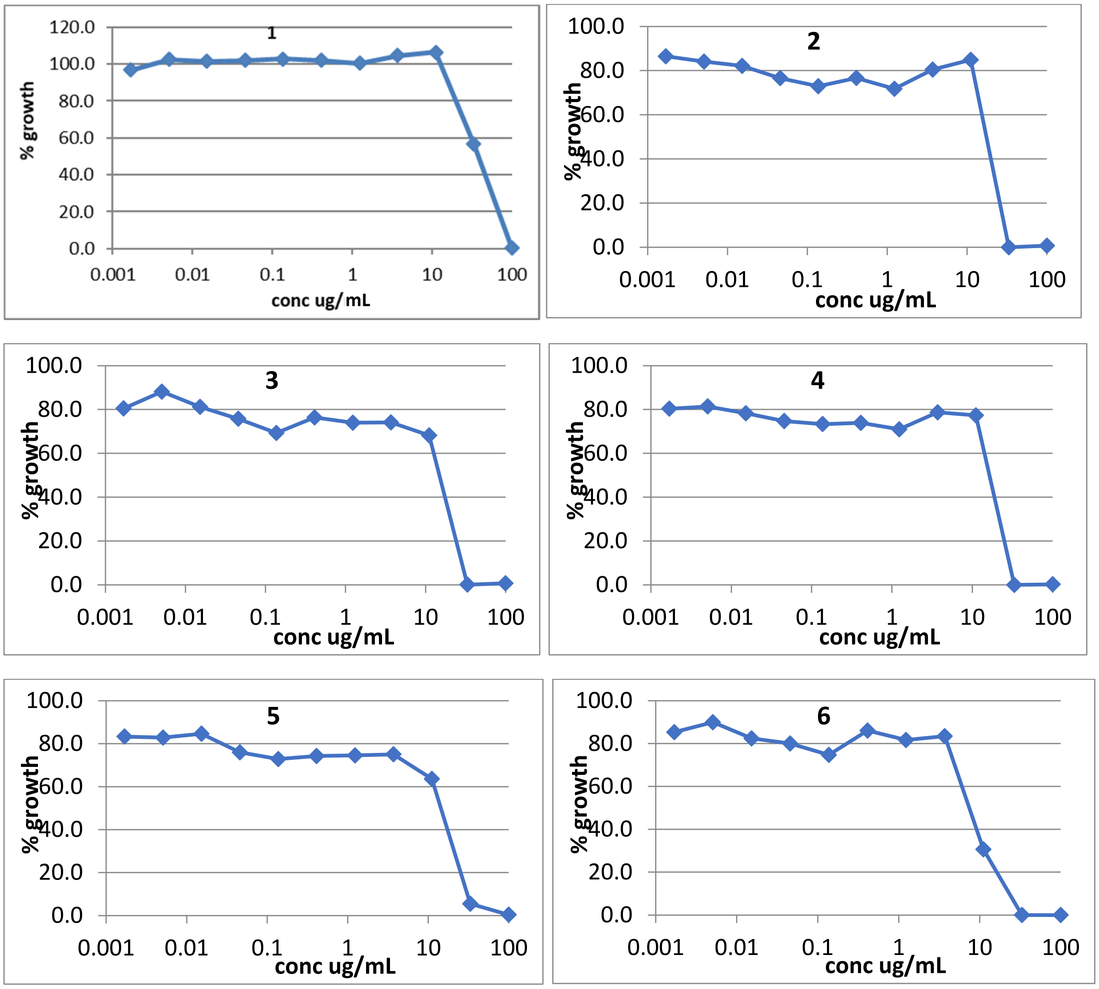

Figure S2: Dose–effect diagrams for determination of cytotoxicity of compounds **2**–**6** against L-6 cells.

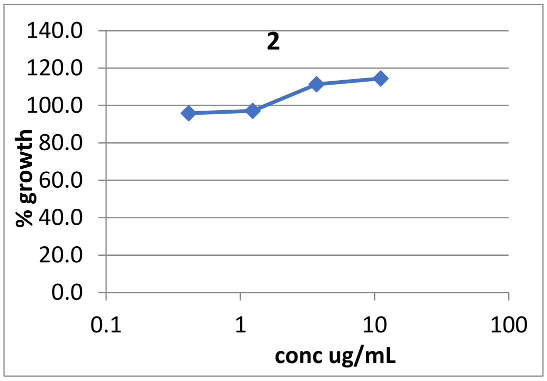

Figure S3: Dose–effect diagram for activity of compound **2** against *Leishmania donovani* intracellular amastigotes.
